# Quantitative monitoring of paramagnetic contrast agents and their allocation in plant tissues via DCE-MRI

**DOI:** 10.1186/s13007-022-00877-z

**Published:** 2022-04-11

**Authors:** Simon Mayer, Eberhard Munz, Sebastian Hammer, Steffen Wagner, Andre Guendel, Hardy Rolletschek, Peter M. Jakob, Ljudmilla Borisjuk, Thomas Neuberger

**Affiliations:** 1 Leibniz-Institute of Plant Genetics and Crop Plant Research (IPK), Corrensstrasse 3, 06466 Seeland-Gatersleben, Germany; 2 Institute of Experimental Physics 5, University of Würzburg, Am Hubland, 97074 Würzburg, Germany; 3 Institute of Experimental Physics 6, University of Würzburg, Am Hubland, 97074 Würzburg, Germany; 4 Huck Institutes of the Life Sciences, The Pennsylvania State University, 113 Chandlee Lab, University Park, PA 16802 USA; 5 Department of Biomedical Engineering, The Pennsylvania State University, 113 Chandlee Lab, University Park, PA 16802 USA

**Keywords:** *Hordeum vulgare*, Contrast agent (CA), Magnetic resonance imaging (MRI), DCE-MRI, Gadolinium DTPA, Vascular bundles, Plant monitoring

## Abstract

**Background:**

Studying dynamic processes in living organisms with MRI is one of the most promising research areas. The use of paramagnetic compounds as contrast agents (CA), has proven key to such studies, but so far, the lack of appropriate techniques limits the application of CA-technologies in experimental plant biology. The presented proof-of-principle aims to support method and knowledge transfer from medical research to plant science.

**Results:**

In this study, we designed and tested a new approach for plant Dynamic Contrast Enhanced Magnetic Resonance Imaging (pDCE-MRI). The new approach has been applied in situ to a cereal crop (*Hordeum vulgare*). The pDCE-MRI allows non-invasive investigation of CA allocation within plant tissues. In our experiments, gadolinium-DTPA, the most commonly used contrast agent in medical MRI, was employed. By acquiring dynamic T_1_-maps, a new approach visualizes an alteration of a tissue-specific MRI parameter T_1_ (longitudinal relaxation time) in response to the CA. Both, the measurement of local CA concentration and the monitoring of translocation in low velocity ranges (cm/h) was possible using this CA-enhanced method.

**Conclusions:**

A novel pDCE-MRI method is presented for non-invasive investigation of paramagnetic CA allocation in living plants. The temporal resolution of the T_1_-mapping has been significantly improved to enable the dynamic in vivo analysis of transport processes at low-velocity ranges, which are common in plants. The newly developed procedure allows to identify vascular regions and to estimate their involvement in CA allocation. Therefore, the presented technique opens a perspective for further development of CA-aided MRI experiments in plant biology.

**Supplementary Information:**

The online version contains supplementary material available at 10.1186/s13007-022-00877-z.

## Background

Progress in magnetic resonance imaging (MRI) opened the possibility to noninvasively assess structure, metabolism and gene expression in living organisms [11, 15, 26, 27, 31, 43, 44]. Different methods for plant MRI have been developed and are now in use and operate with high spatiotemporal resolution and sensitivity [5, 6, 39, 47, 48, 59, 60, 62]. While tremendous advances have already been achieved with these MRI applications, the study of dynamic processes remains a challenge in plant science. The versatility of MRI largely overcomes the limitations caused by the specific tissue structure of plants compared to animals [34], but concepts for CA-aided MRI experiments are still underdeveloped for plant biology.

There are a range of optical methods for determining flow velocities such as dye-tracing approaches [52], delivering high spatial resolution and accuracy. However, they often are limited by the fact that tissues deeply embedded (like transport vessels) are not accessible. Noninvasive approaches include acoustics [30], heat flow [3], X-ray [45] and neutron imaging [2]. Another powerful tool to assess in vivo assimilate allocation is ^11^C- and ^14^C-autoradiography and positron emission tomography (PET) [14, 29]. Also [18F]- and [19F]-fluorinated compounds (like 2-deoxy-2-fluoro-d-glucose or 6-[F18] fluoro-6-deoxysucrose) are used for real time monitoring of translocation as well as for analyzing solute transport, root uptake, photoassimilate tracing, carbon allocation, and glycoside biosynthesis [18, 56, 57]. However, using ionizing radiation raised numerous concerns and appears challenging in practical use.

MRI is capable to overcome some of these limitations by using non-radioactive paramagnetic contrast agents (CAs). For CA-aided MRI experiments, the most commonly used CAs produce either a hypo intense signal in the image (mostly iron-based CAs), or a hyper intense signal, depending upon the paramagnetism of the chemical compound [66]. The right choice of both CA and detection procedure are prerequisite for successful MRI-measurements. Due to the nature of plant tissue with many air pockets and cell walls [34], the use of hypo intense CAs is largely limited.

Gadolinium (Gd) based MRI CAs provide a hyper intense signal and are suitable for plant and (pre-)clinical examinations. In both its chelated and unchelated form, Gd is non-toxic for plants [46]. Since the first experiments with Gd in plant roots [9], the CA has been preferentially used to visualize water transport. Later, Kuchenbrod et al. [33] applied CA and phase contrast flow imaging techniques to quantitatively determine water flow velocities and total volume flow rates in corn vessels. Gussoni et al. [24] successfully used two different CAs (D_2_O and Gd-DTPA) to investigate molecular transport processes in the stem of the morning glory plant (*Pharbitis nil*). For the evaluation of transport across the pericarp in mature grape berries, Mn enhanced MRI (MEMRI) was used by Dean et al. [13]. Their calculation was based on tissue specific longitudinal MR relaxation time (T_1_) maps. An important outcome of these and many other studies was that the transport velocities of the CA could be determined in various organs and tissues of different plant species.

The functional imaging method Dynamic Contrast Enhanced (DCE)-MRI is a CA based (pre)clinical imaging method that provides an efficient solution for a wide range of applications including velocity measurements. The method relies on the fact that the paramagnetic CA instantly alters tissue-specific parameters (mainly the longitudinal relaxation times T_1_). Due to this characteristic change (lowering of T_1_) an improved signal to noise ratio (SNR) can be achieved. The arrival of the CA, the residence time, and the clearance rate of the specific tissue can be monitored. One major challenge is the fact that the CA-effect on the NMR signal is not directly proportional to the concentration of the CA. If the CA concentration is of interest, a so called T_1_-map must be generated at different time points to conduct this quantification. Two important points should be considered when working with plants: first, for plant imaging with microscopic resolutions, the acquisition of a T_1_-map using standard procedures would take hours, thus would be too long for estimating dynamic parameters in living organisms. Second, DCE-MRI is working well in magnetically relative homogeneous tissues, and therefore, as mentioned above, is routinely used in (pre-)clinical studies [19]. As the distribution of the CA within the blood stream is fairly rapid, DCE is mostly based on T_2_* (‘observed’ transverse relaxation time) weighted gradient echo sequences, enabling fast data acquisition. Plant tissues with many air inclusions are unfortunately magnetically heterogeneous [5, 34] and the T_2_* weighted gradient echo sequence based on small flip angles is therefore not feasible. To perform dynamic contrast enhanced imaging in plants, experimental plant biologists need a different robust method with higher sensitivity and better temporal resolution.

Because of the unique structure of plant cells and the much slower flow rate in plant vessels (as compared to animal vasculature), it is extremely difficult to transfer MRI-methods directly from the (pre-)clinical field to plant research. Here we developed a new approach based on a spin echo sequence, which is less sensitive to magnetic inhomogeneities typically found in many plant tissues. Our work builds on the study from Tofts [54] who used a DCE-MRI approach to study the permeability of the blood–brain barrier in humans. Our experiments exemplified how the Tofts’ method can be adapted for in situ applications in plants. The concept and suitability of the new method for experimental plant biology was tested in the crop plant barley (*Hordeum vulgare*). The novel plant-specific approach was named plant DCE-MRI (pDCE-MRI) and aims at bridging the gap between traditional destructive labeling experiments and direct observations on the living plant, a feature rarely achieved so far. The proof-of-principle presented here also highlights potential limitations of the method, which need further future improvement.

## Methods

### Plant material

Barley plants (*Hordeum vulgare,* cultivar Barke) were cultivated in growth chambers under a light/dark regime of 16/8 h at 20/14 °C, light 600 µmol quanta m^−2^ s^−1^ (at ~ 1 m above ears) and a relative air humidity of 60%. The MRI measurement was performed during the main seed filling stage. The stem of the barley plant was cut off with a razor blade and immediately placed into a 5 mm NMR glass tube filled with 0.5 ml nutrient solution containing ¼ Murashige and Skoog medium. Injection of the contrast agent was performed via tubing without touching the setup. The setup was fixed using teflon tape and positioned inside the NMR coil.

### Hardware for magnetic resonance imaging

The experiments were performed on a Bruker Avance III HD 400 MHz 9.4 T NMR spectrometer (Bruker BioSpin, Rheinstetten, Germany) equipped with a 1000mT/m gradient system. For determination of the relaxivity of the CA and the plant MRI experiments a saddle coil with an inner diameter of 10 mm was used.

### Magnetic resonance imaging parameters

For the pDCE experiments multi-slice, T_1_-weighted spin echo images were acquired. The measurement parameters were chosen as following: Repetition time TR = 500 ms, echo time TE = 3.4 ms, field of view FOV = 3.5 × 3mm^2^ with an in-plane resolution of 35 µm, slice thickness 1 mm. Five parallel slices were acquired, separated by 1.75 mm to estimate vertical transport velocities. Using a partial Fourier acquisition scheme allowed us to reduce the measurement time by 25% to 32 s per image.

The reference T_1_-map (one before CA administration and one at the end of the experiment) was a standard saturation recovery slice-selective spin-echo (SE) sequence (TE = 3.4 ms, varying repetition times TR = 0.25, 0.5, 0.75, 1, 2, 4, 6, 8 s). The image resolution, slice number, slice thickness, and slice position were exactly the same as in the T_1_-weighted sequence. The total acquisition time for the reference T_1_ map was 1 h 56 min.

### DCE-imaging method

In order to capture the dynamics of the living plant, a fast imaging protocol was used, similar to the work described by Tofts and Kermode [54]. The timeline for the whole experiment was as follows. After the standard long saturation recovery reference T_1_-map was recorded, eight fast T_1_-weighted images (parameters: see above) were acquired before the CA administration as baseline for the initial T_1_ values (timepoint t = 0). Following the CA administration of 0.5 ml containing 2 mM Gd (resulting in a 1 mM Gd liquid medium solution), a series of fast T_1_-weighted images were collected to monitor the dynamics of the signal change due to the change in T_1_ and hence the CA uptake. Overall, 1672 fast T_1_-weighted data sets were acquired during a period of 14 h and 52 min. Due to the relatively poor SNR of one single image a sliding window scheme was used to average 16 consecutive images maintaining the high temporal resolution. Dynamic T_1_-maps were calculated using the signal change in the T_1_-weighted images and the reference saturation recovery T_1_-map from the beginning. As the T_1_-parameter depends on the concentration of the CA present in the volume, the recording of the dynamics of the T_1_-values enables the calculation of the local concentration over time (see below in Theoretical considerations). At the end of the experiment a second standard long saturation recovery T_1_-map with the same parameters as before was acquired and compared to the last calculated pDCE-T_1_-map (see Additional file 1: Fig. S1).

### Contrast agent and determination of the relaxivity

The contrast agent gadolinium-diethylenetriamine pentaacetic acid (Gd-DTPA, Magnevist®, Bayer) used in this work is based on gadolinium. Gadolinium contains seven unpaired electrons and is therefore strongly paramagnetic. Thus, in the presence of the CA, the relaxation process is accelerated, compared to the natural relaxation process. This leads to a reduction of the longitudinal relaxation time $T_{1}$ and at higher concentrations to a reduction of the transversal relaxation time $T_{2}$ as well. The shortened relaxation parameter $T_{i,1}$ (i = 1, 2) itself is dependent on the initial value $T_{i,0}$ and the concentration c of the CA within the sample [54]:

1 $$\frac{1}{{{ }T_{i,1} }} = \frac{1}{{{ }T_{i,0} }} + c \cdot R_{CA,i} \quad \left( {i = 1, 2} \right)$$

With the relaxivities $R_{CA,1}$ and $R_{CA,2}$, the ability of the CA to shorten the $T_{1}$ and $T_{2}$ at 9.4 T, respectively. The determination of the relaxivities $R_{CA,1}$ and $R_{CA,2}$ of the CA was performed by measuring T_1_ and T_2_ of solutions with different concentrations of the CA in distilled water. The dilutions were produced in the range of 0 to 1 mM. A saturation recovery multi-echo spin-echo-sequence with varying repetition times (TR) was used for the relaxivity measurements. The slope of a linear fit through the measured values of R_1_ = 1/T_1_ plotted over concentration *c* enables the calculation of the relaxivity $R_{CA,1}$. Due to the use of low Magnevist concentrations and a short echo time TE (see below), a reduction of T_2_ was negligible in any of our experiments.

### Data analysis

To determine the translocation velocities of the CA, the concentration of the CA in the living tissue had to be derived from the dynamically measured T_1_-values. The initial standard reference T_1_-map was calculated by an in-house written MATLAB (The MathWorks Inc, Natick, MA, USA) algorithm using a least-squares fit. Before the dynamic T_1_-maps were calculated, motion correction was applied to the T_1_-weighted images using MATLAB to account for the minor movement of the whole plant. The dynamic T_1_-maps of the plant tissue were calculated based on the consecutively acquired T_1_-weighted images. The hereby determined Gd-DTPA concentration value in the plant tissue was used to calculate the vertical transport velocities of the CA: Therefore, the increase of CA concentration in all slices could be monitored and the velocity of the CA along the stem could be derived when taking the different time points of the arrival of the CA in the first and the last slice into account. Furthermore, the distribution of the CA over time perpendicular to the stem (= in each slice) could be assessed.

### Fourier-transform infrared spectroscopy (FTIR) sucrose imaging

To reference the pDCE distribution map to known functional allocation patterns, a set of barley stem cross sections was imaged according to Guendel et al. [22]. After embedding stem sections in Tissue-Tek O.C.T. (Sakura Finetek, the Netherlands), 12 µm thick slices were cut by cryo sectioning and placed on MMI membrane slides (Molecular Machines & Industries GmbH, Germany). Lyophilized sample sections were imaged in the wavenumber range from 3800 to 900 cm^−1^ with a spectral resolution of 6 cm^−1^ and 15× magnification on a Bruker Hyperion 3000 hyperspectral microscope (Bruker Opics GmbH & Co. KG, Germany). Images were recorded using a globar infrared source of a Bruker Invenio S spectrometer and the focal plane array detector set up of the microscope. The entire setup was continuously purged with dry air. Images for sucrose were created and calibrated according to the protocol established by Guendel et al. [22].

## Results

### Experimental model and setup

**Fig. 1:**
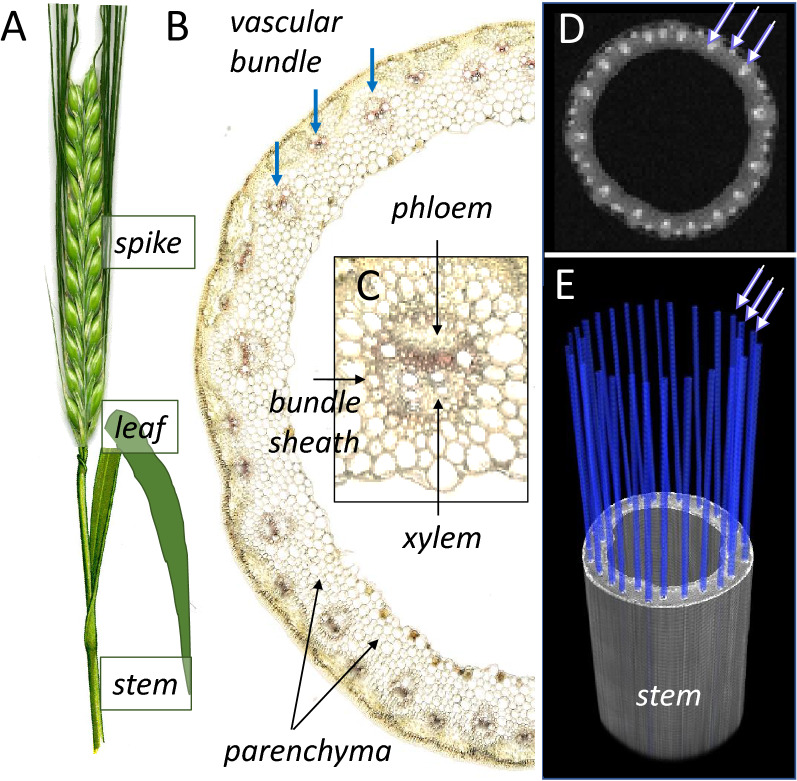
Structure of the stem in barley by light microscopy and MRI. **A** The fragment of the stem with leaf and spike at the top. **B** Light microscopic section through the stem. Numerous vascular bundles of different sizes are clearly visible (arrowed) in the parenchyma tissue of stem. **C** The structure of a single vascular bundle showing the localization of phloem and xylem inside of the vascular bundle. **D** NMR image shows an axial cross section through the stem. **E** NMR-based model showing the 3D arrangement of vascular bundles (blue) inside the stem

Barley was chosen as a plant model for this study not only due to its economic importance as an agricultural crop, but also based on our long experience in experimental studies of its physiology, metabolism, and genetics [7, 37, 50]. Barley (Fig. 1A) belongs to the family of the monocots and has collateral vascular bundles, which comprise both xylem (transporting mainly water/nutrients) and phloem (transporting mainly assimilates). The cryosection of the barley stem shows typical vascular bundles of monocots, where the xylem and the phloem are enclosed by a bundle sheath of parenchyma cells (Fig. 1B and C). Individual vascular bundles are scattered within the ground parenchyma tissue of the stem and are arranged along its longitudinal axis as shown in the MR image in Fig. 1D and the 3D-model in Fig. 1E. The inner space of the stem is filled with air.

**Fig. 2:**
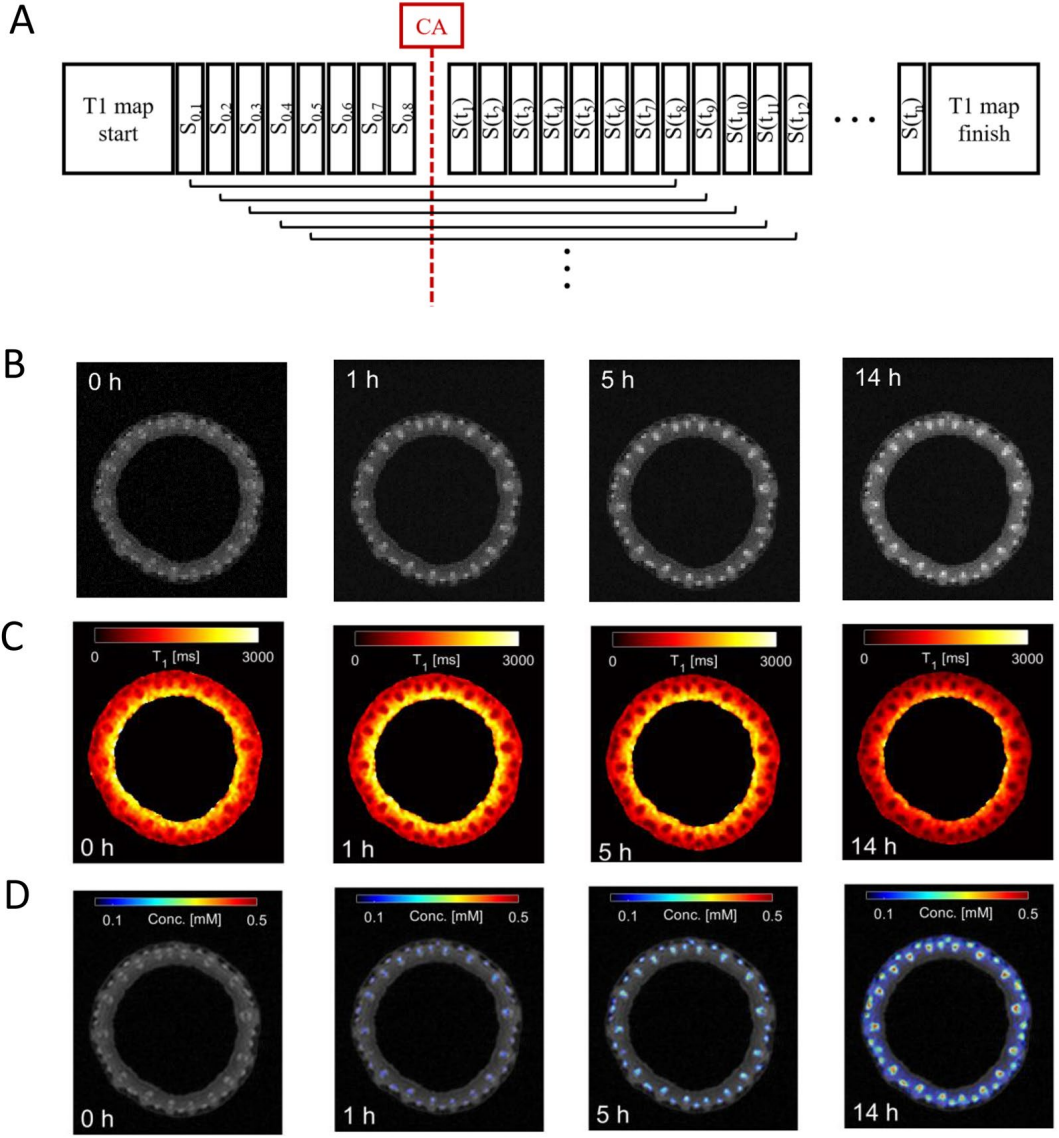
Scheme and application of the pDCE-MRI for monitoring of CA in Barley stem. **A** Scheme of MRI acquisition for pDCE. **B** Brightening of the areas corresponding to the vascular bundles due to gadolinium inflow after the CA administration. The time course is exemplified by the T_1_-weighted images of an axial cross section (lowest of the five measured slices) through the sample. **C** Color-coded images representing the T^1^-maps calculated on the basis of the saturation recovery T^1^-map (shown in Fig. 3A) and the T^1^-weigthed images (shown here in **B**) by using the pDCE method. A decrease of T^1^ over time can be observed. **D** Calculated concentration maps of the contrast agent based on the T1-maps from **C**; background is the 1H-NMR image acquired before CA administration. Accumulation of CA (color-coded) in the areas of the vascular bundles is clearly distinguishable

A typical DCE experiment and its timeline is depicted in Fig. 2A. After recording the reference T_1_-map, eight fast T_1_-weighted images were acquired before the CA administration. Following the administration of the CA, a series of fast T_1_-weighted images were collected to monitor the dynamics of the signal change due to the change in T_1_ and hence the CA uptake. At the end of the experiment a second T_1_-map was acquired.

### Theoretical considerations

As in Tofts' approach, it was assumed that the concentration of the CA in the plant tissue during the first acquisitions remained zero and significant signal changes were not caused by instrumental fluctuations. As mentioned above, a standard saturation recovery reference T_1_-map was acquired before and ~ 15 h after CA administration. As no significant changes of T_1_ within the dormant tissue in the two maps were observed, a good system stability could be assumed.

In the following, the calculation of the dynamic T_1_-maps is elucidated. A reduced T_1_ leads to a higher NMR-signal S in a T_1_-weighted image. This dependency can be described for every voxel by:

2 $$S\left( {TR, T_{1} } \right)\sim M_{0} \left( {1 - e^{{ - \left( {TR/T_{1} } \right)}} } \right) \cdot e^{{ - TE/T_{2} }}$$

(M_0_ is the equilibrium magnetization; TR and TE are the repetition and the echo time; T_1_ and T_2_ the longitudinal and transverse relaxation times).

Equation 3 is valid for images acquired without CA (S_0_, signal from all the timepoints before the CA reaches the imaging plane) reference T_1_-weighted images; T_1,0_, same as T_1_ in Eq. 2) and for images acquired after CA administration (Eq. 4; T_1,1_, shorter than T_1,0_). T_2,0_ and T_2,1_ are the respective T2 times.

3 $$S_{0} \sim M_{0,0} \left( {1 - e^{{ - \frac{TR}{{T_{1,0} }}}} } \right) \cdot e^{{ - TE/T_{2,0} }}$$

4 $$S_{1} \sim M_{0,1} \left( {1 - e^{{ - \frac{TR}{{T_{1,1} }}}} } \right) \cdot e^{{ - TE/T_{2,1} }}$$

Generating the ratio x of both signals and exploiting $M_{0,0} = M_{0,1}$ and including Eq. (1) yields:

5 $$x = \frac{{S_{1} }}{{S_{0} }} = \frac{{M_{0,1} \cdot \left( {1 - e^{{ - \frac{TR}{{T_{1,1} }}}} } \right) \cdot e^{{ - TE \cdot \left( {\frac{1}{{{ }T_{2,0} }} + c \cdot R_{CA,2} } \right)}} }}{{M_{0,0} \cdot \left( {1 - e^{{ - \frac{TR}{{T_{1,0} }}}} } \right) \cdot e^{{ - TE \cdot \frac{1}{{{ }T_{2,0} }}}} }} = \frac{{1 - e^{{ - \frac{TR}{{T_{1,1} }}}} }}{{1 - e^{{ - \frac{TR}{{T_{1,0} }}}} }} \cdot e^{{ - TE \cdot c \cdot R_{CA,2} }}$$

The determination of the transverse relaxivity yielded $R_{CA,2} = \left( {4.60 \pm 0.52} \right)\frac{{{\text{Hz}}}}{{{\text{mM}}}}$ (see below), resulting in $e^{{ - TE \cdot c \cdot R_{CA,2} }} > 0.98$ for concentrations $c < 1 {\text{mM}}$ and an echo time $TE = 3.4 {\text{ms}}$. Hence, this factor can be assumed as nearly constant during the experiment and will be neglected in the further considerations. Equation (5) can now be solved for T_1,1_

6 $$T_{1,1} \left( {TR, T_{1,0} ,x} \right) = - TR\left[ {\ln \left( {1 - x\left( {1 - e^{{ - \frac{TR}{{T_{1,0} }}}} } \right)} \right) } \right]^{ - 1}$$

and the new dynamic $T_{1,1}$ for every voxel within the DCE-image can be calculated. $T_{1,1}$ is dependent on the TR, the initial value $T_{1,0}$ (taken from the reference T_1_-map), and the ratio of the signal intensities x in each voxel. As mentioned in Eq. 1, the concentration c of the CA can be calculated at each time point with the now known parameter $T_{1,1}$:

7 $$c = \frac{{T_{1,0} - T_{1,1} }}{{T_{1,0} *T_{1,1} }}*\frac{1}{{R_{Gd - DTPA, 1} }}$$

Unlike other studies utilizing DCE-MRI, these equations can be written in this simplistic form since a standard SE sequence was used, i.e., approximations for small flip angles are not necessary.

Thus, the pDCE method provides the means to perform an accelerated calculation of the T_1_-values. Furthermore, it enables the monitoring of the translocation characteristics of the applied CA in situ in the living plant. Nevertheless, distinct general restrictions of the method should be considered by evaluating the images. It is common in DCE experiments that noise or movement can affect the calculation of a correct T_1_-value, since the ratio $x$ (see Eq. 5) depends on the measured signal amplitudes at different time points.

### Phantom experiment findings

The longitudinal relaxivity of the CA at 9.4 T was estimated from the concentration series as described above:

$$R_{ Gd - DTPA,1} = \left( {3,79 \pm 0,04} \right)\frac{1}{{\text{mmol*s}}}$$

This value can be compared to relaxivities determined at different field strengths. Sasaki [51] found a value of 4.79 (1/mmol*s) at a field strength of 1.5 T and 4.50 (1/mmol*s) at a field strength of 3 T in water phantoms containing dilutions of Gd-DTPA.

The transverse relaxivity was estimated from the concentration series to $R_{CA,2} = \left( {4.60 \pm 0.52} \right)\frac{{{\text{Hz}}}}{{{\text{mM}}}}$. This T_2_ effect can be neglected in our experiment as discussed above.

### Identification of vascular tissues involved in the allocation of contrast agent

Example images of the MR experiment are shown in Fig. 2. Over the course of the experiment the CA enters the plant sample as evidenced by the increasing signal intensities in the sample (Fig. 2B). Especially in the vascular bundles, an increasing signal intensity was observed. The calculation of the DCE-T_1_ maps based on the initial saturation recovery T_1_ map and the dynamic T_1_-weighted images revealed the distribution of the CA across the plant stem (Fig. 2C). Once the CA arrived in the imaged slice, a decrease in T_1_ could be observed over the rest of the experimental time. While tissue further away from the vascular bundles showed only a moderate decrease, a substantial decrease in T_1_ was observed in the vascular bundles (reduction of up to 70%). We conclude that the DCE-T_1_-maps distinguished the active vascular tissue strands from the surrounding plant tissue due to characteristic decreases of their T_1_-values during the experiment. The second saturation recovery T_1_ map, acquired right after the pDCE experiment (~ 15 h after CA administration), showed similar T_1_ values when compared to the last pDCE reconstructed T_1_ map (Additional file 1: Fig. S1). This similarity confirmed the validity of the method. Based on the DCE-T_1_-maps, concentration maps of the CA were calculated by using Eq. (7). Vascular regions were characterized by the highest local accumulation of CA as shown in the color-coded image (Fig. 2D).

### Evaluation of local concentration of the contrast agent in the plant tissue during the experiment

**Fig. 3:**
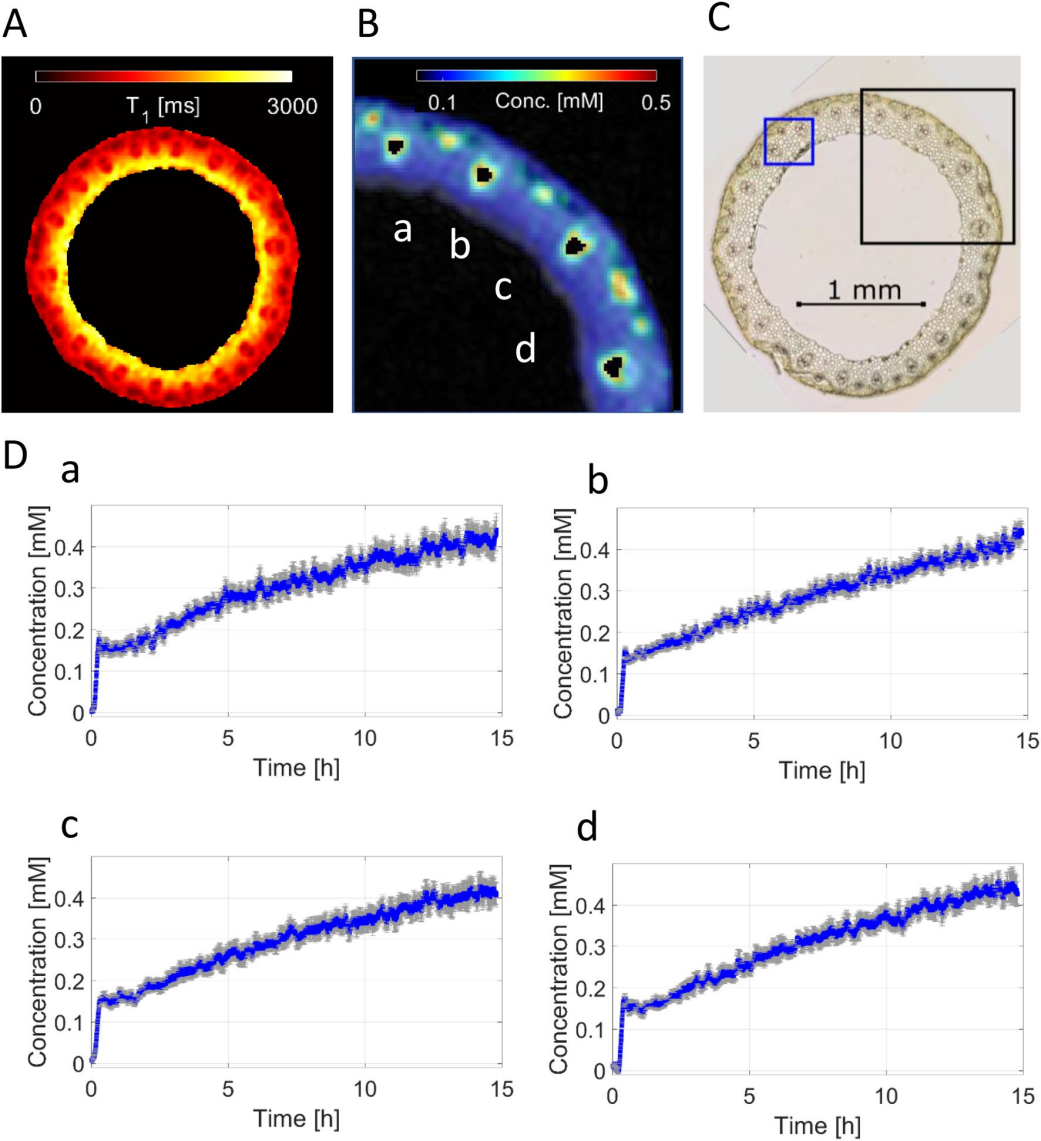
Concentration of CA in four individual vascular bundles of barley stem. **A** Color-coded image representing the standard saturation recovery T_1_-map (cross section through the stem) before CA administration. **B** Fragment of the concentration map after 14 h monitoring showing the four regions of interest at the lowest slice (ROIs, in black). The temporal courses of CA concentration are correspondingly depicted in **D**. **C** Histological image of a slice analyzed by pDCE-MRI (dissected after the monitoring experiment). **D** The time course of the CA concentration in selected vascular bundles (*a*–*d*)

The dynamic intake of the CA in an imaging plane is displayed in Fig. 3. Four randomly chosen vascular bundles taken from the upper right part of the stem (see black box in the histological slide Fig. 3C) are depicted as black ROIs in Fig. 3B. Due to the sliding window technique, the temporal resolution of 32 s per dataset could be preserved. All four ROIs show a similar behavior. After an initial short baseline with no CA present, a relative rapid increase of signal amplitude and therefore drop of T_1_ which means increase of local CA could be observed in the vascular bundles (Fig. 3D). This initial increase of CA concentration to about 0.15 mM was followed by a steady increase of the CA for the remaining experiment. After 15 h an average CA concentration of $\left( {0.43 \pm 0.01} \right) {\text{mM}}$ was reached which was still lower than the 1 mM of the stock solution. Areas away from the vascular bundles showed only minimal signal change and therefore only minimal CA uptake at the end of the experiment.

### Determination of the vertical transport velocities of the contrast agent

**Fig. 4:**
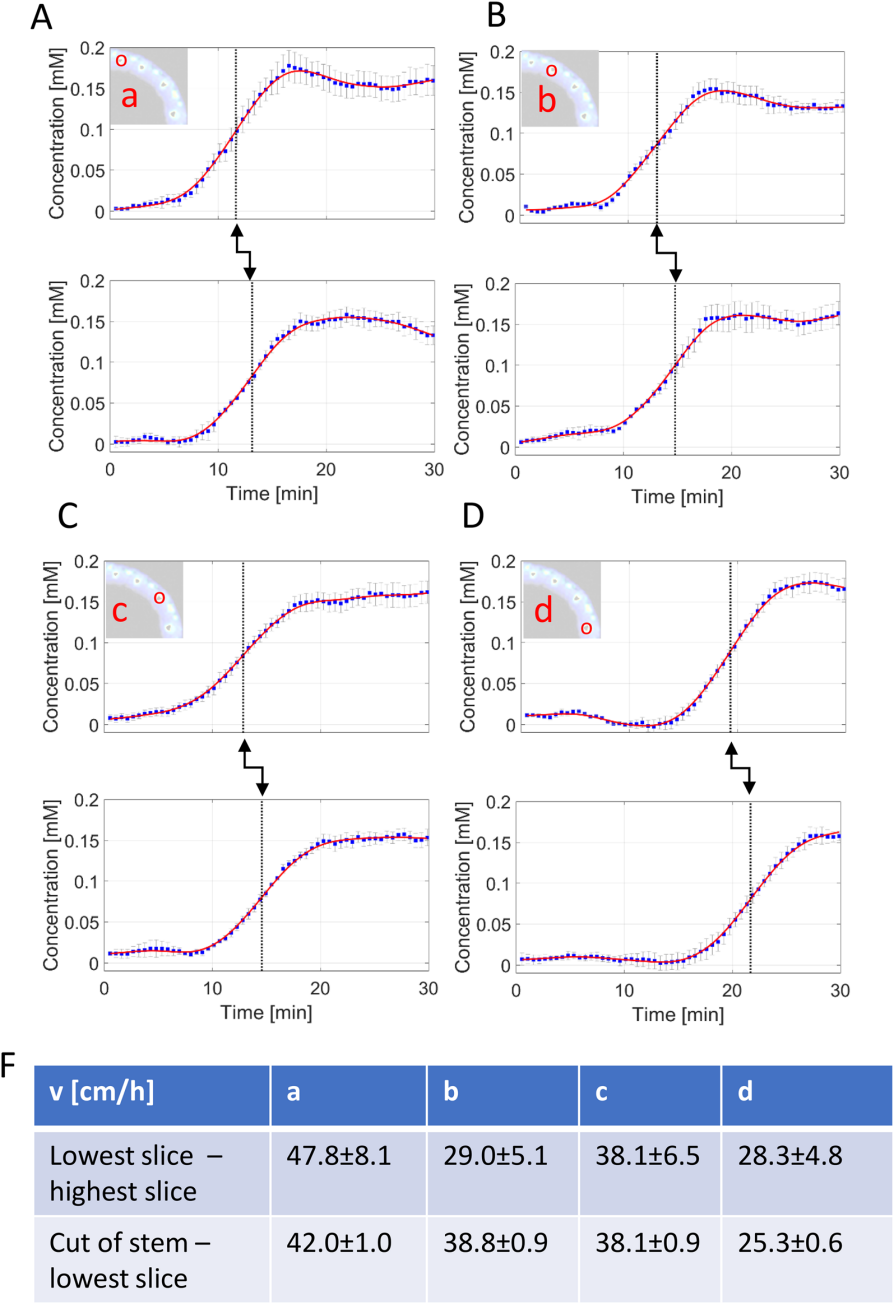
Estimation of CA velocity in individual bundles. **A** The monitoring of individual vascular bundle at the lowest slice over the entire experiment (upper panel). Simultaneous monitoring of the same vascular bundle at the highest slice (low panel). The distance between the lowest and highest slice was 1.1 cm. The temporal delay of the CA arrival at highest slice is clearly distinguishable (arrowed). This is exploited to calculate the vertical transport velocities of the CA along the vascular bundles. **B**–**D** Analogous monitoring data are shown for other bundles (*b*, *c* and *d*, as pictured in Fig. 3B). **E** The velocity value as estimated using monitoring data

We further applied pDCE-MRI to estimate CA velocities along the vascular bundles. Velocity in the vertical direction was estimated by two different approaches.For each of the two methods the timepoint of arrival of the CA in the imaging slice had to be determined. For this, the concentration data points of the first 30 min after CA administration were considered (see Fig. 4) which display the arrival and increase of CA. The precise time of CA arrival was assumed as the turning point of the CA increase which was determined by the numerical calculation of the root of the second time derivative of the concentration curve.

In the first approach, the distance between the lowest imaging plane and the highest imaging plane of the multi slice experiment was utilized to calculate the vertical CA velocities. The two imaging planes were positioned as far apart as the homogeneity of the RF-resonator allowed. The distance between the planes was 1.1 cm. The upper plot of each figure (Fig. 4A–D) is the CA time course of the lowest imaging slice, and the lower plot is the CA time course of the highest imaging slice. The black arrow like structure shows the delay of the arrival of the CA in the highest slice compared to the lowest slice. The calculated vertical velocities of the CA are displayed in the first row of the table in Fig. 4E. Velocities ranged between 28.3 and 47.8 cm/h.

In the second approach, the velocity was calculated by dividing the distance from the cut of the stem to the middle imaging slice, by the time from CA administration to the time it took for the CA to arrive. In this straightforward method, the distance from the bottom of the stem to the center of the RF-resonator (corresponding to the location of the center slice) was measured with calipers. Furthermore, the positioning of the RF-resonator in the center of the magnet was confirmed beforehand in water phantom experiments. The second row in Fig. 4E displays the velocities calculated with this method in the four vascular bundles depicted in Fig. 3B. The velocities ranged from 25.3 to 42.0 cm/h. The stated errors resulted from the standard deviation of the concentration values within a ROI.

### Dynamic imaging of contrast agent in stem tissues

**Fig. 5:**
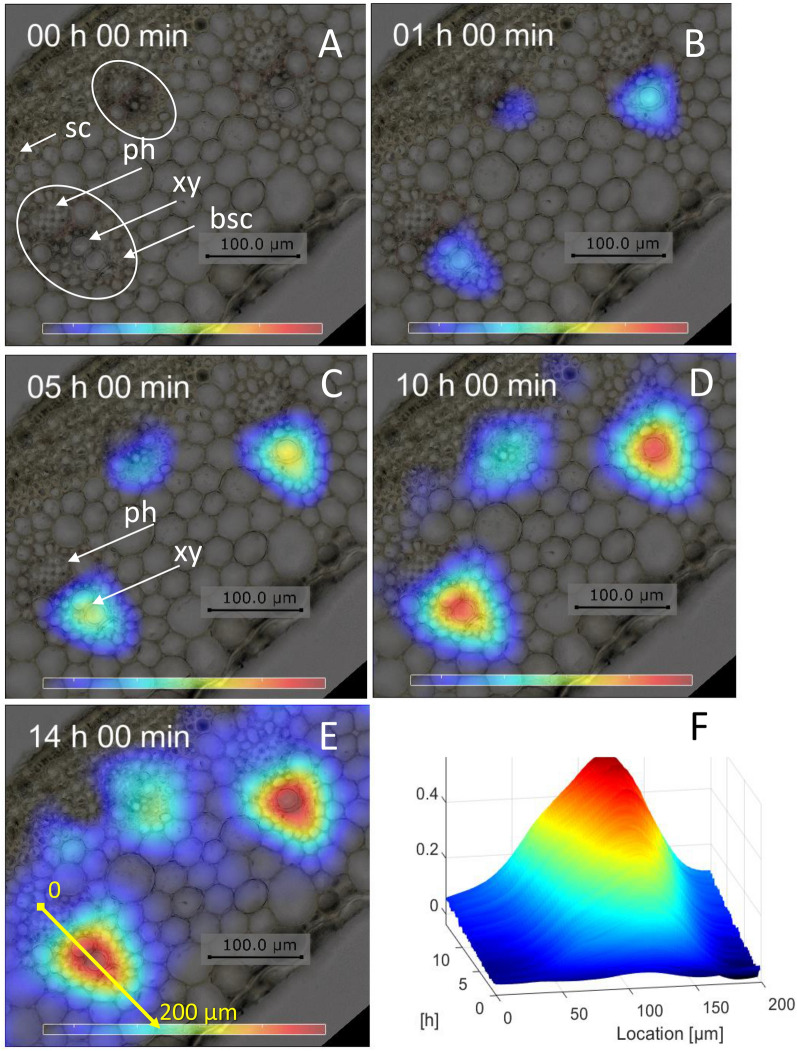
Observation of the horizontal movement of the CA within one axial slice. **A**–**E** A section of the histology image of stem (after monitoring, see blue box in Fig. 3C), overlaid by the corresponding concentration images (pDCE-MRI) for different time points can be seen. The accumulation and spreading of the CA around the vascular bundles are detectable (**F**) Representation of the temporal accumulation of the CA along the yellow line marked in the image of **E**. ph: phloem; xy: xylem; bsc: bundle sheat cells; sc: sclerenchyma

Figure 5A displays a histological section of the stem including several vascular bundles. The phloem, the xylem, and the bundles sheath cells (bsc) of a representative vascular bundle are depicted within the white oval of the first image. The colored overlay represents the CA concentration at the beginning of the experiment, at 1 h, 5 h, 10 h, and after 14 h. The CA arrives first in the xylem about 1 h after CA administration; Fig. 5B. A slight increase of CA concentration could be observed inside the xylem during the next 4 h of monitoring (Fig. 5C; Additional file 2: Movie S1). No CA was detected in the phloem of the same vascular bundles. In contrast, the phloem shows the highest levels of sucrose, as visualized by FTIR imaging in cryosections (Additional file 1: Fig. S2).

Later, the CA diffuses slowly into the bundle sheath cells closest to the xylem tissue and some CA accumulation was seen in the phloem and parenchyma tissues after 10 h of experimental time (Fig. 5D). Some traces of CA were detected in the trunk parenchyma at 14 h (image Fig. 5E; Additional file 3: Movie S2). Profiles of the time course of CA accumulation along the yellow arrow shown in the 14 h image are shown in Fig. 5F (Additional file 3: Movie S2).

These data indicate that the CA moved a certain distance perpendicular to the main vertical strand. Such leakage of the CA from the vascular vessel was rather unexpected as Gd-DTPA was reported not to pass through cell membranes [16]. In the current state, the resolution of the presented method is not sufficient to determine the velocity of this movement (horizontal velocity of the CA). Nevertheless, our observation clearly shows that CA can move outwards of the vascular bundles. The nature of horizontal transport (diffusion or controlled transport) as well as the ultrastructural features underlying CA leakage from vascular filaments remains to be studied.

## Discussion

This proof-of-principle aims to support method and knowledge transfer from medical research to plant science. We described an approach to apply the principle of DCE-MRI methodology to plants. The novel procedure enables the non-invasive monitoring of CA allocation in plant tissues with high spatial and temporal resolution and sensitivity. Based on our experiments on barley, we discuss the potential advantages of the plant DCE-MRI, but also pinpoint to its apparent limitations.

### Advantages and limitations of plant dynamic contrast-enhanced MRI (pDCE-MRI)

Our experiments demonstrate how to improve the temporal resolution of T_1_-mapping to enable the dynamic quantitative measurement of the local concentration of the CA’s concentration by adapting the approach from Tofts [54]. In our experiment, we acquired the T_1_-map before administration of Gd-DTPA and detected CA repeatedly after distinct incubation time via consecutive T_1_-weighted images of known T_1_-weighting. Using this procedure, the signal enhancement (monitored in T_1_-weighted images) enables the calculation of dynamic T_1_-maps of plants.

A major advantage of pDCE-MRI is that the method provides both qualitative and quantitative data on CA allocation and allows to calculate the concentration of the CA in the relevant plant tissues. This information can be used, for example, to identify the vascular arrangement and to estimate their involvement in CA allocation. One might detect regions that accumulate CA but which cannot be identified as a vascular volume in individual T_1_-weighted images. Therefore the CA might provide information on vascular arangment below image resolution (qualitatively). The direct and unambiguous identification of active transport tissue due to the fast and high-resolution T_1_-maps of plant organs is one of the major features of the proposed pDCE-MRI method. Here we demonstrate that numbers of vascular bundles in the stem of barley could be visualized and monitored simultaneously over a long period of time.

The pDCE-MRI method can be combined with other CAs, like manganese ions [12], cobalt ions [21], or iron particles [4]. For the evaluation of transport across the pericarp in mature grape berries, Mn was used [13] and calculation was also based on T_1_-maps. Our new approach could find applications aside from the biological imaging in further research fields, e.g. to study the transport characteristics in porous media either based on T_1_-weighted imaging [40] or via the calculation of T_1_-maps [4]. The choice of CA should be carefully considered, as for example, some CA have a stronger effect on the transverse relaxation time T_2_, thus reducing the amount of signal acquired in regions where the CA accumulates. Such negative contrast was created with Co^2+^-ions in a biofilm-mediated ion exchanger [21].

The features of the various tracers including Gd are not yet fully understood [10, 17, 68]. More confidence in the interpretation of experimental data could be achieved by integration of pDCE-MRI with other techniques based on e.g. fluorescent tracers [67], PET [28], radioisotope-imaging [53], or ^13^C-MRI [38] in the future. The combined use of these methods could provide additional information about the structure and composition of tissues in respect to metabolite allocation.

In animals, Gd-DTPA can easily be injected to perform DCE-MRI while this is not an option in plants. Gd-DTPA is only taken up if the apoplast (and/or symplast) is opened by cutting the tissues (e.g., roots or stem). Therefore, this remains one fundamental limitation for Gd-DTPA based pDCE-MRI applications in plants.

In general, measurements using pDCE-MRI could be disturbed by the displacement of tissue. Especially during long time monitoring, plant “movements” (e.g. in response to light) should be considered. In its current state, pDCE-MRI is a promising approach, but further development is needed for reliable applications in routine work.

### Relevance of plant DCE-MRI for experimental biologists

While the MR imaging platform provides a variety of reliable flow measurement methods, most of these methods directly measure the flow of water and rarely measure the velocity of molecules, which are allocated along with the water flow [32, 50, 58, 65]. The dynamic of water flow certainly affects but does not necessarily explain the allocation of CAs.

Differently sized CAs are translocated via the vascular system with distinct velocities (which differ from that of water). In addition, they target different tissues in the plant [8, 41, 67]. By using fluorescent tracers Wang et al. [63] showed that small molecules such as the Lucifer Yellow LYCH (molecular mass Mr = 0.5 kDa) could easily permeate. Whereas small proteins (Mr = 15–30 kDa) are generally less mobile [64] and movement of large molecules (e.g., dextran Mr = 500 kDa) is even more restricted within plant tissues [1].

The molecular diameter of the Gd-DTPA is approximately 8.2 Å, and its molecular weight is 938 amu [42]. The sucrose molecule has a molecular diameter of 9 Å [49] and a molecular weight of 342 amu. Therefore, Gd-DTPA and sucrose are very similar in size and weight. Hall et al. [25] reported an average velocity of ^14^C-labelled assimilates in castor bean plants of approximately 82 cm/h [25], we measured Gd-DTPA velocities in the barley stem from 25.3 to 42.0 cm/h in vertical direction (Fig. 3). However, it should be pointed out again, that sucrose is actively transported into and out of the cells, and thus the contrast agent most certainly will not behave and be transported in the same way as sucrose. More importantly, Gd-DTPA does not pass through cell membranes [16]. Other compounds are translocated with a velocity between 0.5 cm/h and 6 cm/h, as measured by the real-time radioisotope imaging system (RRIS) for mineral compounds in *Arabidopsis thaliana* [53]. Thus, our experiments demonstrate that pDCE-MRI in combination with the paramagnetic CA allows to monitor flow velocity in the range of centimeters per hour and is thus appropriate for observation of the activity of the vascular system and/or detection of tissues involved in molecular trafficking in living plants.

Investigation of the vascular bundles shows that the CA is first detectable in small regions of tissues. These regions correspond to vessel elements of the xylem. Each vessel element is surrounded by smaller, living, highly active xylem parenchyma, all encased in the bundle sheath cells. This is supporting tissue which regulates transport into and out of the vessel elements. Next the detection in the supporting tissue and finally in the parenchyma tissue (surrounding of the vasculature) was possible. This behavior can be explained by the following. First, the spike which is located at the top of the stem, consists of numbers of developing grains supported by the husk. The spike develops a strong water sink due to the transpiration activity of the husk and the water absorption by the growing grains. The delivery of water occurs mainly through the xylem. The xylem contains vessels of large diameter (Fig. 3). During the feeding of CA into the cut stem, the CA travels with water into the xylem vessels and is transported with the water flow upwards toward the spike. Correspondingly, the CA is first detected in the xylem. The high velocity (up to over 40 cm/h) and the direction of the flow measured using pDCE-MRI corresponds well with the upstream route of water delivery toward the spike [50]. In contrast, the phloem vessels are filled with sucrose, with concentrations up to about 0.3 M [22]. One of the main differences between phloem and xylem is that the phloem is alive and has active cell contents. Sucrose and other transported substances must be moved through the sieve plate between sieve elements. Despite the close vicinity of the phloem and xylem inside of the vascular bundles, no CA was detectable in these narrow thin-walled phloem tubes during the first 5 h of observation. Thus, phloem vessels are sharply delineated from xylem, and there is no substantial exchange of CA between these two tissues. This fact [55, 61] has been argued to be vital to facilitate the counter directional flow of water towards source organs like leaves and the osmotic off-load of assimilates towards sink organs (e.g. seeds), which would be disturbed if substantial uncontrolled water leakage between xylem vessels and phloem tissue was possible throughout the vascular structure. Similar xylem and phloem associated distribution patterns have also been found for various metabolite groups essential for distinct metabolic functions as recently presented [23]. This distinct functional compartmentalization, phloem assimilate transport and xylem water transport, essentially follows the same general direction in the stem towards the spike, but is counter directional in leaves (i.e. source leaves, supplying assimilates to other parts of the plant).

Due to the high sensitivity of pDCE-MRI, we were able to detect the leakage of the CA from the vasculature into the stem, which was a rather unexpected event. While plants have no transporters for CAs and gadolinium is a non-membrane-permeable substance, question were raised on how this might occur? From an anatomical perspective, this observation could be explained by the influx of Gd-CA from the vascular tissue to the ground parenchyma (along the horizontal direction). Important to notice is, that using highly sensitive LA-ICP-MS (Laser ablation with inductively coupled plasma mass spectrometry) the CA was detected in roots, stems, and leaves of garden cress exposed to gadolinium in a study by Lingott et al. [36]. Gadolinium was predominantly detected within the leaf veins and the leaf tip, whereas in the stem it was colocalized with the transport of water towards the leaves.

Generally, such an allocation is expected to be rather slow and sub-cellular structures involved in the process are less studied. Either symplastic (cytoplasm-to-cytoplasm) and/or apoplasmic pathways could be involved in this transport, but the mechanism and the driving forces of the observed horizontal CA allocation await further investigation. We believe that further refinement of the pDCE-MRI method will make it possible to quantify these processes in the future and can be easily combined with earlier elegant feeding approaches in plant science [35].

## Conclusion

The biggest advantages of MRI are its non-invasive nature and its soft tissue contrast. It provides an in-vivo platform to investigate living organisms including plants. While MRI with its many diverse well-established methods is the preferred technique for non-invasive imaging in medical science, only some of these procedures are available for plant research. In this work we applied the concept of DCE-MRI to the cereal crop model barley and demonstrate how this method can be adjusted for plant applications. In its current iteration the method allows non-invasive quantification of large molecule transport within the stem and determines both, its flow velocities and concentration changes in an in-vivo setup. An unexpected dispersal of the CA in the horizontal direction was discovered but needs more clarification in future experiments. The high spatial and temporal resolution of the established procedure opens new ways to study plant dynamics. Overall, the presented method of pDCE-MRI will open new perspectives for further future developments of CA-aided MRI experiments in plant biology. ​

## Supplementary Information

**Additional file 1.** Supplementary Figure S1: Comparison of the final T1-maps and Supplementary Figure S2: Distribution of gadolinium versus sucrose in barley stem.

**Additional file 2.** Supplementary movie 1: pDCE monitoring of the CA inflow over the first 5h.

**Additional file 3.** Supplementary movie 2: pDCE monitoring of the CA inflow over the entire observation time.

## Data Availability

The datasets used and/or analyzed during the current study are available from the corresponding author on reasonable request.

## Abbreviations

CA: Contrast agent
FOV: Field of view
FTIR: Fourier-transform infrared spectroscopy
Gd-DTPA: Gadolinium-diethylenetriamine pentaacetic acid
LA-ICP-MS: Laser ablation with inductively coupled plasma mass spectrometry
MEMRI: Mn enhanced MRI
MRI: Magnetic resonance imaging
pDCE-MRI: Plant Dynamic Contrast Enhanced Magnetic Resonance Imaging
ROI: Region of interest
RRIS: Real-time radioisotope imaging system
SE: Spin echo
SNR: Signal to noise ratio
T_1_: Longitudinal relaxation times
T_2_: Transverse relaxation time
T_2_*: ‘Observed’ transverse relaxation time
TE: Echo time
TR: Repetition time
