# Supplementary material for: Quantitative monitoring of paramagnetic contrast agents and their allocation in plant tissues via DCE-MRI
Source: Plant Methods. 2022 Apr 11;18:47. doi: 10.1186/s13007-022-00877-z (PMC8996644; doi:10.1186/s13007-022-00877-z)
Supplement: Supplementary file 1 — Additional file 1. Supplementary Figure S1: Comparison of the final T1-maps and Supplementary Figure S2: Distribution of gadolinium versus sucrose in barley stem. [file 13007_2022_877_MOESM1_ESM.pdf]

Supp. Figure S1 & S2

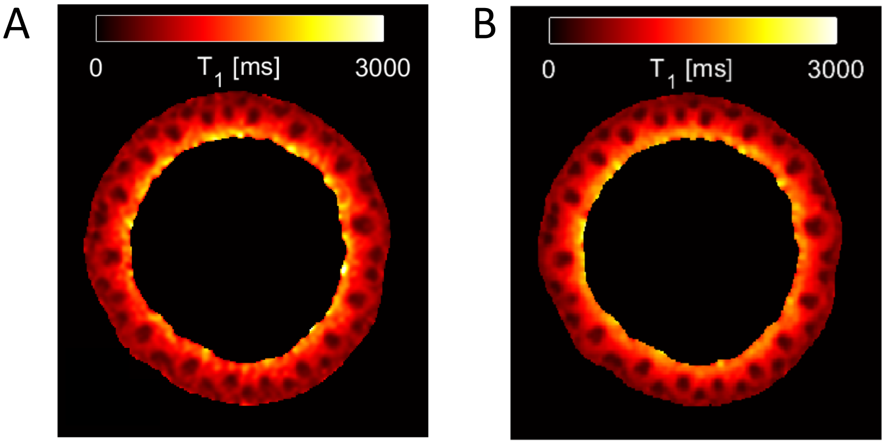

Supplementary Figure S1. Comparison of the final T_1-maps: (A) The final T1-map calculated with the pDCE method using the reference T_1-map acquired before CA administration and the last T_1-weigthed image after just under 15 h. (B) The final T_1-map recorded with the saturation recovery control measurement at the end of the experiment. To large extent, the good match of the T_1-values in both maps is observed and evident for the reliability of the T_1 values calculated with the pDCE method.

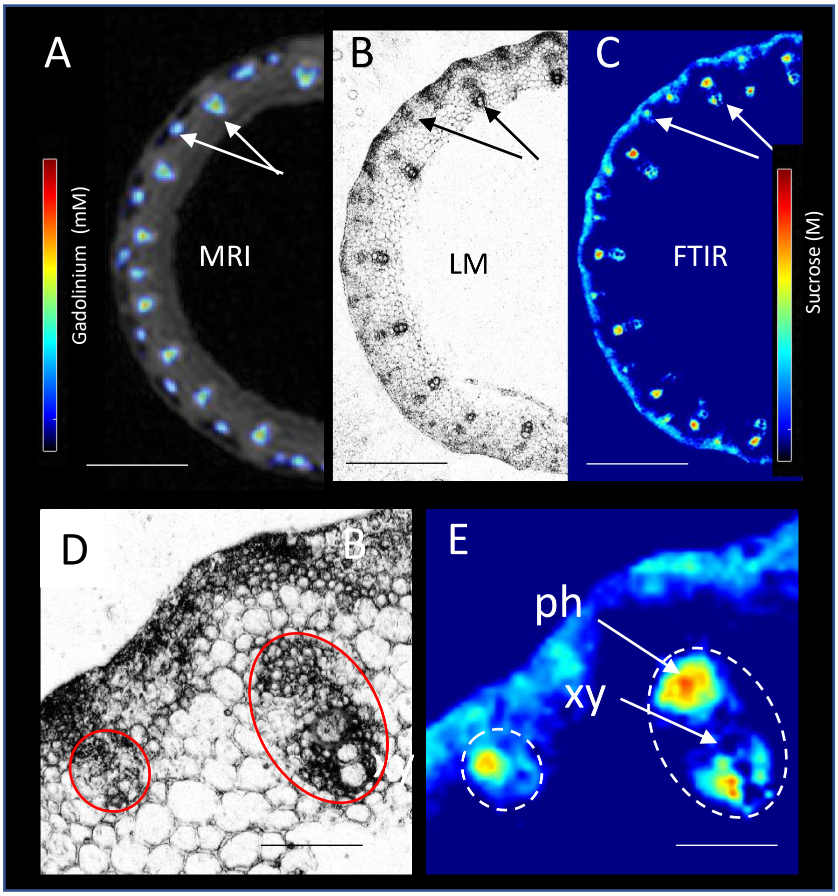

Supplementary Figure S2. Distribution of gadolinium versus sucrose in barley stem. (A) Visualization of gadolinium concentration by non invasive pDCE MRI; (B) Structure of the stem in cryosection as used for FTIR measurements; (C) Quantitative sucrose map through the stem by FTIR microimaging. (D, E) Enlargement from B and C shows, that the highest level of sucrose is seen in the phloem region (ph) of the vascular bundle, but much lower level in the xylem region (xy). FTIR and LM images show exactly the same section (B and C, as well as D and E) , whereas MRI image (A) is a virtual section of stem. This demonstrates a clear structural division of vascular bundles according to transport tissue types.
